# Risk Factors of Metabolic Syndrome among Polish Nurses

**DOI:** 10.3390/metabo11050267

**Published:** 2021-04-23

**Authors:** Anna Bartosiewicz, Edyta Łuszczki, Małgorzata Nagórska, Łukasz Oleksy, Artur Stolarczyk, Katarzyna Dereń

**Affiliations:** 1 Institute of Health Sciences, Medical College of Rzeszów University, 35-959 Rzeszów, Poland; eluszczki@ur.edu.pl (E.Ł.); kderen@ur.edu.pl (K.D.); 2 Institute of Medical Sciences, Medical College of Rzeszow University, 35-959 Rzeszow, Poland; nagorska@ur.edu.pl; 3 Orthopaedic and Rehabilitation Department, Medical University of Warsaw, 02-091 Warsaw, Poland; loleksy@oleksy-fizjoterapia.pl (Ł.O.); artur.stolarczyk@wum.edu.pl (A.S.)

**Keywords:** metabolic syndrome, cardiovascular diseases, health behaviors, obesity, nurses, risk factor

## Abstract

The metabolic syndrome, also known as syndrome X or the insulin resistance, is defined by the World Health Organization as a pathologic condition characterized by abdominal obesity, insulin resistance, hypertension, and hyperlipidemia. Both all over the world and in Poland, there is a shortage of nurses; most of those employed are in the pre-retirement age. However, the requirements in this profession and the patient’s right to care at the highest level remain unchanged and do not take into account the poor condition or age of working nurses, so special attention should be paid to the state of health in this professional group. There is an emphasis on the importance of the adopted attitude toward health and the resulting behaviors, such as regular weight control, following dietary recommendations, regular physical activity and participation in preventive examinations. The aim of the study was to assess the frequency of the occurrence of the metabolic syndrome, its individual components and determining the factors influencing its development in Polish nurses. The research conducted among the nurses in question included DXA (Dual Energy X-ray Absorptiometry) measurements, assessment of glucose concentration, lipid profile, blood pressure and a questionnaire survey. Almost half of the surveyed nurses have metabolic syndrome, which significantly increases the risk of developing cardiovascular diseases or diabetes. After multivariate analysis, it was found that being overweight and obesity were significant factors influenced the MS (metabolic syndrome) occurrence among Polish nurses. Being overweight increases the chances of MS occurrence 8.58 times in relation to BMI (Body Mass Index) <25, obesity increases the chances of MS occurrence 8.085 times in relation to BMI <25, and obesity class II/III increases the chances of MS occurrence 16.505 times in relation to BMI <25. Preventive and supportive measures for this professional group are needed.

## 1. Introduction

The issue of appropriate health behaviors and self-care is increasingly being taken up by scientific communities around the world [1,2,3]. Scientists emphasize the importance of the adopted attitude toward health and the resulting behaviors, such as regular weight control, following dietary recommendations, regular physical activity and participation in preventive examinations [4,5,6,7,8]. Metabolic syndrome is a complex of interrelated risk factors for the development of cardiovascular disease (CVD) and diabetes. These factors include elevated levels of glucose, triglycerides, blood pressure, low HDL-cholesterol (High-density lipoprotein cholesterol) and abdominal obesity [9,10]. MS is recognized as the cause of morbidity and the scourge of mortality not only in developed countries but also in underdeveloped countries. According to the data provided, in Poland and worldwide, more and more people meet the criteria for its diagnosis, more importantly, at an increasingly younger age [11,12]. There are many definitions of MS but since 2009, the IDF (International Diabetes Federation) and AHA/NHLBI (American Heart Association/National Heart, Lung and Blood Institute) have developed a common position that defines how to recognize MS. The diagnosis of MS requires the presence of at least three out of five factors. They include the following: an abnormal waist circumference specific for a population or country (>94 cm in men and >80 cm in women); blood pressure—systolic ≥130 and/or diastolic ≥85 mm Hg (or treatment hypotensive); elevated fasting glucose ≥100 mg/dL(or hypoglycemic treatment); elevated triglycerides concentration ≥150 mg/dL (or lipid-lowering treatment); and reduced HDL–C (high-density-lipoprotein cholesterol) cholesterol fraction of <40 mg/dL in males and <50 mg/dL in females [13,14]. The complex disorders in the carbohydrate and lipid economy, arterial hypertension and visceral obesity shown above prove the disturbing condition of people who suffer from it, and it can have a negative impact on their health in the future as it can lead to reduced productivity and increased sickness absences [15,16]. Many studies have estimated the prevalence of MS. Researchers from the United States (U.S.) examined 8814 people aged 20 years and older. The results presented that the prevalence of MS increased from 6.7% among participants aged 20–29 years to 43.5% for participants aged 60–70 [17]. In another study conducted in China with 1206 participants, the incidence of MS was 26.7%, and the prevalence of diabetes and hypertension were 4.3% and 38%, respectively [18]. One study conducted in Riyadh showed that the overall prevalence of MS was 35.3%. The age-adjusted prevalence, according to the standard European population, was 37%. Low HDL–cholesterol influenced the majority of MS risk factors [19]. In the study of Saudi soldiers aged 20–60 years, the age-adjusted prevalence of MS was 20.8%. The most common component in the study population was abdominal obesity (33.1%), followed by high serum triglycerides (32.2%) and raised systolic blood pressure (29.5%). A total of 71% of participants exhibited at least one criterion for MS [20].

The problem that many countries are currently facing is demographic change in societies with high demand for care services; considering the shortage of nursing staff, this can be challenging for many countries [21,22,23,24]. Nurses are a key element in the health care system, representing approximately 59% of all health workers worldwide [25,26]. Working under extreme stress, long hours of shift work and the need to make difficult decisions are part of the daily routine of many nurses but are also the factors that result in the development of many health problems [27]. It is a situation where it is much easier to make a mistake and react less adequately to the needs resulting from everyday work [28,29,30]. Today, around one million registered nurses worldwide are over 50 years of age, which means that a third of the workforce may reach retirement age within the next 10–15 years. The age of Polish nurses has been on the increase and now it is about to reach 52 years, but also the number of nurses between the ages of 50 and 70 is more than four times the number of nurses aged 26–50 [25,31]. Due to the decreasing interest in the profession among young people, as well as the emigration of already qualified staff, the lack of natural replacement of generations in the profession needs to be tackled [25,32,33,34]. In order to maintain an employment balance, it seems necessary to promote a healthy lifestyle among nurses who, despite the approaching retirement age, are still professionally active. At the same time, it is worth emphasizing that the requirements regarding the professional competences and the patient’s right to care at the highest level remain unchanged and do not take into consideration the poor conditions or ages of working nurses. [35,36]. Because of their biomedical education and interdisciplinary competences, nurses should demonstrate a particularly high level of pro-health awareness and motivation, which, in turn, can be implemented for the sake of own health, as well as that of the patients [37,38,39]. The Brazilian study, based on the data of over a thousand nurses, indicates a high level of MS prevalence in this professional group and its association with work environment, stress and occupational burnout [40]. Additionally, Ribeiro indicates that anxiety and depression [41], as confirmed by the evidence available in the literature, and a stressful work environment are associated with the incidence of cardiovascular disease and the development of MS [42,43,44]. The justification for undertaking the research is the specificity of nurses’ work, the unquestionable burden of duties and sometimes the necessity to work in several entities simultaneously. All this can lead to negligence of one’s own health and, consequently, to the development of serious diseases, including MS.

The aim of the study was to evaluate the frequency of the occurrence of the metabolic syndrome and its individual components and determining the factors influencing its development in Polish nurses.

## 2. Results

### 2.1. Characteristics of the Study Group

A total of 108 women aged 49 to 55 were surveyed. The mean age of the respondents was 52.29 ± 3.85 years. Almost half of women worked in hospital and 48.15% had one shift work; 44.44% of respondents had a master’s degree in nursing. The descriptive characteristics of the study group are presented in Table 1.

The values of individual parameters in the total of 108 nurses are presented in the Table 2. The average BMI was 27.56 ± 5.87 with mean A/G ratio 1 ± 0.21.

In Table 3, the variables are presented as a category. The majority of all respondents were nurses that were overweight (*N* = 40; 30.04%). In the study group, 2 women (1.85%) were underweight, 35 subjects (32.41%) had normal body mass, and 31 people (28.7%) were obese. A total of 39.82% of respondents had high blood pressure. Two women had diabetes and almost 1/3 pre-diabetes. Three fourths of the women had elevated cholesterol (68.52% raised LDL cholesterol and 10.19% HDL cholesterol below standard). Almost half of the participants had elevated triglycerides (Table 3).

#### 2.1.1. The Health Behavior Inventory

The Health Behavior Inventory (HBI) questionnaire assessed the health behavior of the respondents. A total of 48 out of 108 respondents (44.44%) had an average level of health behavior, 35 (32.41%) had a low level of health behavior, and 25 (23.15%) had a high level of health behavior (Table 4).

#### 2.1.2. International Physical Activity Questionnaire (IPAQ)

The IPAQ (International Physical Activity Questionnaire) questionnaire assessed the general level of physical activity of the study participants. A total of 43 out of 108 respondents (39.81%) had insufficient activity, 35 respondents (32.41%) had high activity, 28 respondents (25.93%) had sufficient activity, and 2 respondents (1.85%) left the questionnaire unfilled (Table 5).

### 2.2. The Findings

Table 6 presents the occurrence of selected factors considered risk factors for MS.

#### 2.2.1. Univariate Risk Analysis for the MS

Logistic regression models (separate for each of the analyzed features) showed that significant (*p* < 0.05) predictors of the odds of occurrence of the MS are the following:

- Age.
- Master’s degree in nursing.
- Overweight.
- Obesity.
- Obesity II/III grade.

Triglycerides > 150 mg/dL:

- Age.
- Overweight.
- Obesity.
- Obesity II/III grade.

HDL < 50 mg/dL:

- High activity in IPAQ.

Glucose ≥ 100 mg/dL:

Logistic regression models (separate for each of the analyzed features) showed that none of the analyzed variables are a significant predictor of the chance of its occurrence (*p* > 0.05).

Blood pressure > 130/85 mmHg:

- Master’s degree in nursing.
- Specialization.
- Activity high in IPAQ.

Abdominal obesity:

- Age 51–55 years.
- Age over 55.
- Very good self-esteem of health condition.
- Participation in preventive examinations.
- Overweight (Table 7).

#### 2.2.2. Multivariate Risk Analysis for the Metabolic Syndrome

Odds ratios for influencing components were calculated (Table 8). The logistic regression model showed that significant (*p* < 0.05) independent predictors of the odds of MS occurrence are as follows:

- Overweight.
- Obesity.
- Obesity class II/III.

Triglycerides > 150 mg/dl:

The logistic regression model showed that significant (*p* < 0.05) independent predictors of the odds of MS occurrence are as follows:

- Overweight.
- Obesity.

HDL < 50 mg/dL:

The logistic regression model showed that none of the analyzed features is a significant independent predictor of the chance of its occurrence (as all *p* > 0.05).

Glucose ≥ 100 mg/dL:

The logistic regression model showed that significant (*p* < 0.05) independent predictors of the odds of its occurrence are as follows:

- Work in the clinic.
- Specialist course.

Blood pressure > 130/85 mmHg:

The logistic regression model showed that a significant (*p* < 0.05) independent predictor of the chance of its occurrence is as follows:

- Activity high in IPAQ.

Abdominal obesity:

The logistic regression model showed that a significant (*p* < 0.05) independent predictor of the chance of its occurrence is as follows:

- Overweight (Table 8).

**Figure 1:**
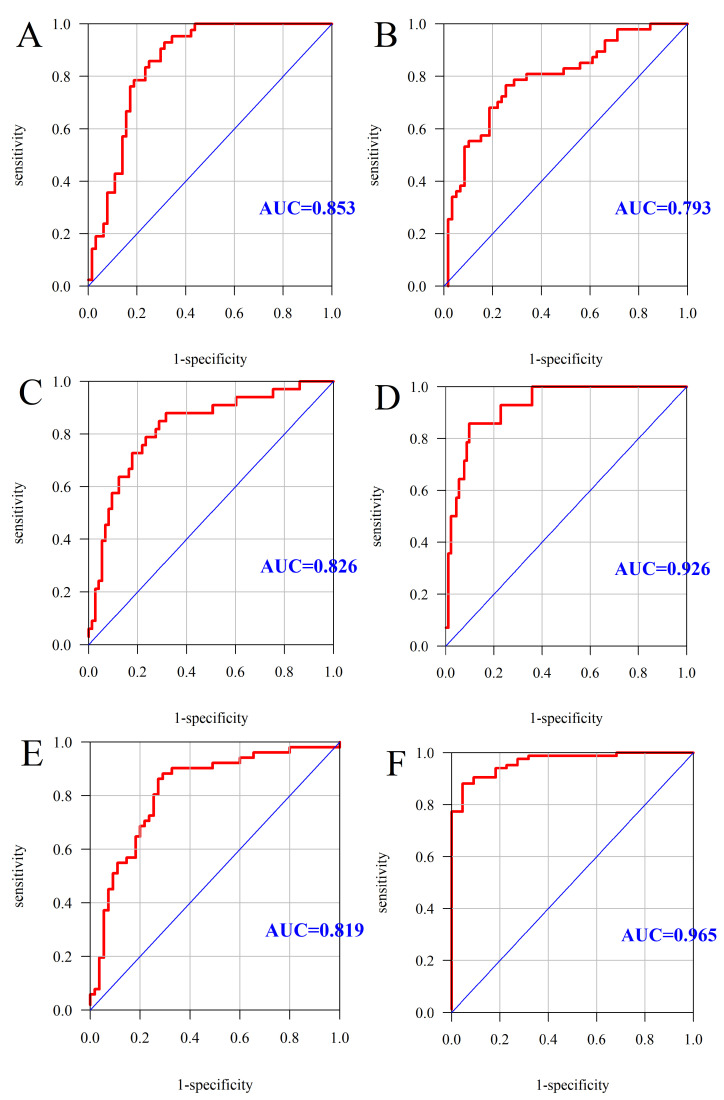
ROC curve and AUC value: MS (**A**), triglycerides > 150 mg/L (**B**), HDL < 50 mg/dL (**C**), glucose ≥ 100 mg/dL (**D**), blood pressure > 130/85 mmHg (**E**), abdominal obesity (**F**). AUC—Area Under the Curve (measure of diagnostic accuracy).

The ROC analysis (receiver operating characteristic curve) results are presented in the Figure 1.

## 3. Discussion

This is the first Polish study to investigate factors associated with MS in this professional group. This issue seems to be important due to the increased needs in the health care of aging societies, the problems of the education system, the staff immigration and the nursing shortage in Poland. A global problem that has a huge impact on health policy is the demographic changes in societies with a simultaneous shortage of medical staff, including nurses. In addition, over a million of those currently employed are 52 years old or more, so in the next 10 years they will retire. The nursing shortage is a challenge for many countries, hence in a situation where rich countries are struggling with a shortage of nurses, it is worth asking about the situation in Poland, especially since it is one of the countries where relative spending on health care is the lowest among all European Union members (6.3% of GDP—Gross Domestic Product); OECD—Organization for Economic Cooperation and Development report) [45]. Poland also has a very low rate when it comes to the number of nurses employed directly in the care of patients: currently, it is 5.2/1000 inhabitants, with the EU (European Union) average of 9.4/1000 [26,45].

Currently, the average age of a statistical Polish nurse is 53, so they will experience numerous diseases. The interest of young people in this profession is waning, and the number of nurses in the pre-retirement age is four times higher than the number of young people entering the profession, so we are dealing with a generation gap. Due to the above, various decisions are being made to keep nurses in the health care system, despite retirement age. The Main Chamber of Nurses, in cooperation with the Ministry of Health, has prepared an offer for the nurses to obtain the right to convalescent leave for this professional group [26,46].

This study showed that 38.9% of nurses had MS. It may cause serious health consequences in the future and can influence their health conditions, the practicing of the profession and, consequently, the efficiency of the health care system. MS is considered a risk factor for coronary artery disease as, confirmed by the research of many authors [47,48,49]. People with MS are twice as likely to die and three times more likely to have a heart attack or stroke compared with people without the syndrome [50]. The researchers presented relation with other disorders, including fatty liver disease, sleep disordered breathing or chronic kidney disease [51,52].

The association between menopausal transition and the incidence of CVD is well known and described in the literature. The rise in CVD risk is connected to the significant hormonal changes, especially estrogen deprivation, at the time of menopause [51]. A decrease in estradiol levels can influence the development of metabolic disorders, such as hypertension, dyslipidemia, and increased central adiposity, which are observed to be cardiovascular risk factors [53]. In the study conducted in China, women who had been menopausal for <1 year compared to women 2–3 years after menopause had higher CVD prevalence and higher TG levels [54]. In addition, authors showed that 10 to 14 years after menopause, there is an increased risk of higher TG. Time since menopause may correlate with MS or obesity [55]. The average age of the participants in our population may indicate that most of the women were of postmenopausal age. According to the American Heart Association (AHA), coronary heart disease is more common in older men than in older women [56]. Testosterone, the major sex hormone for men, is also demonstrated to exert cardioprotective function. The decrease in hormone levels may play a significant role in the development of CVD in men and women, but some authors showed that hormone replacement therapies have not yet shown a significant benefit with respect to cardiovascular health [57].

In the study of Conceição das Merces et al. [40], 24.4% of examined nurses had MS, but in their study, 52.2% of the population were under 35 years old. The average age in our study was 52.2 years. In a Scottish cross-sectional study, obesity prevalence was high across all occupational groups including nurses (25.1%), other healthcare professionals (14.4%), non-health-related occupations (23.5%), and unregistered health care workers who had the highest prevalence of obesity (31.9%) [58].

As demonstrated by our results, among nurses aged 51–55, 51.5% had MS, and 45.8% of nurses had MS in ages over 55. Our results suggest that the MS prevalence is positively associated with age. The logistic regression models show that age 51–55 increases the chance of MS occurrence 4.051 times in relation to the age of 50 years; age over 55 increases the occurrence 3.279 times. These findings are in line with the study which evaluated prevalence and factors associated with MS in nurses in Brazil [40]. It is similar to other investigations [41,59,60]. In the study conducted in Botswana, 34% of the health workers had MS, 28.7% were obese, and 27.3% were overweight. The female gender was found to be strongly associated with MS [61].

In our model, a master’s degree in nursing reduces the chances of MS occurrence by 64.9% in relation to secondary education. In the study of Li et al., women with a higher level of education had lower prevalence of MS [62]. This means that level of education affects the occurrence of MS. This could be connected with the higher knowledge about the prevention of MS, health education and knowledge on preventative measures. Even though health care workers are considered to be well informed about the etiology and risks of being overweight and obesity, studies conducted in most countries confirm the high prevalence of these pathologies in these groups [63]. According to Mohanty et al., studies conducted in most countries, including the U.S.A., Mexico, South Africa and Nigeria, have consistently found them to have disproportionately higher risks of being overweight and obesity compared to the general population [64].

After multivariate analysis, it was found that being overweight and obesity were significant factors that influenced the prevalence of MS among Polish nurses. Being overweight increases the chances of MS occurrence 8.58 times in relation to BMI <25, obesity increases the chances of MS occurrence 8.085 times in relation to BMI <25 and obesity class II/III increases the chances of MS occurrence 16.505 times in relation to BMI <25. In our study, 65.74% had excessive body weight, of which 70% women with obesity class II and III had MS. In the study performed in the U.S.A., the odds of MS rose with being overweight (OR = 4.7) and obesity (OR = 30.6) in relation to having normal body weight [65]. It is noteworthy that an increased body weight has the most influence. In another study, the risk of CVD mortality was significantly higher in overweight people with MS, but a non-higher risk was observed among the healthy overweight population [66].

The logistic regression model showed that significant (*p* < 0.05) independent predictors of the odds of triglycerides and abdominal obesity occurrence were being overweight and obesity. Being overweight increased the chances of triglycerides >150 mg/dL occurrence by 7.625 times in relation to BMI < 25, and obesity increased the chances of this occurrence by 7.095 times in relation to BMI < 25. Being overweight increased the chances of abdominal obesity occurrence 297.419 times in relation to BMI < 25. The effect of obesity on triglyceride levels is well described in the literature [67,68,69].

During multivariate analysis, the following factors influenced glucose levels: work in the Primary Health Care increased the chances of glucose ≥ 100 mg/dL occurrence 15.376 times compared to work in the hospital, and the specialist course increased the chances 70.043 times. The influence of these factors may indicate the level of stress that accompanies the work of nurses. Working in an ambulatory may generate a higher level of stress than working in a hospital ward. Further studies should include the influence of different types of workplaces (units/departments) on stress levels. Similarly, a specialized course may affect stress generated during work, which is confirmed by the findings of other researchers [70]. The literature shows that stress may lead to an increase in glucose levels [71]. The relation between stress and glucose levels includes interference with carbohydrate metabolization following various stressors, potentially leading to insulin resistance [72].

The influence of physical activity on blood pressure has been described in detail in the literature [73,74,75]. High physical activity reduced the chances of blood pressure > 130/85 mmHg occurrence by 75.3% in relation to insufficient activity. Among the studied nurses, 23.15% had a high level of physical activity.

There are also a number of potential limitations of the study that need to be taken into account when interpreting the results. This study was limited in geographic scope and should be repeated among a larger sample and in more regions. Additionally, response bias, such as social desirability, is common in self-reported questionnaires. It might have led to underestimation or overestimation of the present results. Another study limitation is the lack of food intake data. Traditional Polish food and eating habits may have influenced the diet of women aged between 50 and 55 years and, thus, affected the development of MS. Being that the study is cross-sectional, the causality and temporality issues should not be considered.

## 4. Materials and Methods

### 4.1. Study Participants

The research was conducted in the first quarter of 2020 among the nurses from the Subcarpathian region willing to participate in the project called The health condition of Polish nurses. The invitation to participate in the study was posted on the website of the regional Nursing Chambers after obtaining the president’s consent. The nurses interested in participating in the research could download all the information about the planned measurements from the website, including the consent to participate in the study, the questionnaire templates, as well as the contact details of a university employee in order to arrange the exact date and time of the measurements. The participation in the research was completely free and voluntary. The participants were assured that the measurements would be anonymous. The following recruitment criteria were used: professionally active nurses, aged 45–55, with no symptoms of infection within the last 2 weeks, not aware of having health issues and willing to participate in the project. The study included the assessment of body mass composition with DXA, the assessment of glucose concentration and lipid profile, the measurement of blood pressure and a research survey on health behavior and undertaking physical activity, and the socio-demographic data of the surveyed nurses. In total, 153 nurses applied to participate in the study; 41 did not meet the inclusion criteria due to incomplete data, and 2 more measurements were excluded. Finally, the results of 108 nurses were included in the study and subjected to statistical analysis.

### 4.2. Measurement

Height was measured three times with an accuracy of 0.1 cm by means of a Seca 213 portable stadiometer. The measurements were made under standard conditions—barefoot in an upright position. The mean value of three measurements was used in the analyses. The measurements were assessed by DXA. The GE Healthcare Lunar iDXA scanner, based on dual energy X-ray absorptiometry, was used for the study. Abdominal obesity was assessed as the ratio of android and gynoid fat mass (A/G ratio) calculated automatically by the Lunar DXA device software (GE Healthcare, Madison, WI, USA). The boundaries of the regions of interest for determining regional body composition were defined by the software manufacturer:

- The android region was defined by the pelvis cut line (lower boundary), above the pelvis cut line by 20% of the distance between the pelvis and neck cut lines (upper boundary), and arm cut lines (lateral boundaries);
- The gynoid region was below the pelvis cut line by 1.5 times the height of the android region (lower boundary), above the lower boundary by twice the height of the android region (upper boundary), and the outer leg cut lines (lateral boundaries).

The A/G ratio was defined as the ratio between the fat percentage in the android (central) regions of interest and that in the gynoid (hip and thigh) regions of interest.

The measurements were carried out in appropriate conditions (the room temperature allowed to conduct the measurements in underwear) by an employee authorized to operate the DXA device, after informing the participants about the conditions of the study and signing an individual consent to participate in it.

Blood pressure measurement:

Systolic blood pressure (SBP) and diastolic blood pressure (DBP) were measured in the morning after participants rested for 5 min in a sitting position, with their backs supported, feet on the floor, and right arm supported, with the elbow at heart level. An appropriate cuff was selected for each participant’s arm circumference. A Welch Allyn 4200B-E2 instrument (Aston Abbotts, UK) was used to measure SBP and DBP. For the analysis, the values of systolic and diastolic pressure were used as the average of three measurements of consecutive measurements.

Laboratory tests:

All clinical laboratory tests were performed in the morning at the Innovative Research Center of the University of Rzeszów after a 9–12 h night fast of the participating nurses. The capillary whole blood for the tests was collected by a registered nurse using aseptic and antiseptic procedures. The total cholesterol, low-density lipoprotein (LDL) cholesterol, high-density lipoprotein (HDL) cholesterol, triglycerides, and glucose level were measured using the Alere Cholestech LDX Lipid Profile (Alere San Diego, Inc., San Diego, CA, USA) cassettes. The Cholestech LDX instrument’s optics were checked by the operator at the start of each clinic using the manufacturer supplied optics check cassette. All the maintenance was performed in accordance with the manufacturer’s instructions. The Internal Quality Control (IQC) checks were performed twice a week using the two levels of manufacturer-supplied control materials. The device was calibrated each day before it was used.

Criteria for defining clinical parameters:

Metabolic Syndrome: Presence of at least three out of five factors, including the following:

- The abnormal waist circumference specific for the population or country (>80 cm in women).
- The blood pressure of systolic ≥ 130 and/or diastolic ≥ 85 mmHg (or treatment hypotensive).
- The elevated fasting glucose ≥ 100 mg/dL (or hypoglycemic treatment).
- The elevated triglycerides concentration ≥ 150 mg/dL (or lipid-lowering treatment).
- The reduced HDL–C (high-density-lipoprotein cholesterol) fraction < 40 mg/dL in males and <50 mg/dL in females.

Body Mass Index:

- 17–18.49 = underweight.
- 18.5–24.99 = correct body weight.
- 25–29.99 = overweight.
- 30–34.99 = 1st degree obesity.
- 35–39.99 = 2nd degree obesity.
- >40 = grade III obesity.

Blood pressure:

- Normal blood pressure: 120–129 mmHg/80–84 mmHg.
- Normal high pressure: 130–139 mmHg/85–89 mmHg.
- Grade 1 hypertension: 140–159 mmHg/90–99 mmHg.
- Grade 2 hypertension: 160–179 mmHg/100–109 mmHg.
- Grade 3 hypertension: ≥180 mmHg/≥110 mmHg.

Fasting glucose:

- Less than 70 mg/dL—hypoglycemia.
- 70 to 99 mg/dL—normal glucose level.
- 100 to 125 mg/dL—elevated glucose levels—pre-diabetes.
- ≥126 mg/dL at least two measurements—diabetes mellitus [12,13,14,76].

Questionnaires

The basic research tool was a research survey consisting of three parts concerning the following: socio-demographic data, health behavior and the frequency of undertaking physical activity of the surveyed nurses.

- Socio-demographic data concerned such aspects as the workplace, education, having additional qualifications, the nature of the work, participation in preventive examinations, self-assessment of health condition and the occurrence of chronic diseases among the surveyed.
- The assessment of health behaviors was carried out by means of the standardized Health Behavior Inventory (HBI) questionnaire, which is a form of self-report containing 24 statements about various types of health-related behaviors. The respondents answered the questions according to five categories, namely almost never, rarely, from time to time, often, almost always, and they were assigned points from 1 to 5. Then, the obtained points were summed up. The general index of the severity of health-related behaviors, measured with the HBI scale, ranges from 24 to 120 points. The higher the score, the greater the intensity of the declared health behaviors. The overall result of the HBI was converted into sten according to the standards (separate for men and women) provided in the key to this questionnaire. The results in sten 1–4 are low, results in sten 5–6 are average, and results in sten 7–10 show high intensity of the specific health behavior [77].
- The level of physical activity was assessed on the basis of a standardized questionnaire of physical activity—International Physical Activity Questionnaire (IPAQ—Polish version). The IPAQ questionnaire allows you to assess the general level of physical activity of the person completing it. Physical activity is divided into three types: intense, moderate and walking. Each of these activities is expressed in the weekly number of minutes you spend on it. The total score of the IPAQ (total physical activity) is expressed in MET units; the more of them, the higher the activity. The standards have been developed for IPAQ to interpret the obtained result [78].

### 4.3. Quality Control

In order to ensure the efficiency and correctness of the measurements performed, a research strategy was developed. The three-person team of university employees was responsible for supervising the measurements, directing the respondents’ traffic to the appropriate laboratories, receiving questionnaires and coding them with the measurement results. The research survey and the raw measurement data have been double-checked to guarantee authenticity and accuracy, including effective data logic checks and editing checks. The database was built with the use of Excel software and the data were entered twice by two data managers to guarantee accuracy and integration.

### 4.4. Statistical Analysis

The analysis of the influence of variables on a dichotomous (two-state) variable was performed using the method of one-way logistic regression. The analysis of the independent influence of many variables on the dichotomous (two-state) variable was performed using the multivariate logistic regression method. The multivariate regression analysis took into account all the variables listed in the first column in Table 8. In both cases, the results are presented in the form of ORs (odds ratios). The quality of the multivariate models was assessed using the ROC curves and the areas below them.

A significance level of 0.05 was adopted in the analysis; all p values below 0.05 were interpreted as showing significant relationships. The analysis was performed in the R program, version 4.0.2 (accessed on 17 August 2020) [79].

## 5. Conclusions

These results show and confirm the evidence presented in the literature on the influence of excessive body mass in the development of MS. Obesity and deficits in health behaviors cause a risk to nurses’ health. What is more, it is possible that it limits their efficiency in treating patients. The situation calls for the intensification of the undertaken solutions. In order to minimize the presence of MS among nurses, therefore, some interventions, such as the improvement of working conditions, monitoring of worker health and safety with diagnostic procedures and medical referrals, diet and physical activity programs, are necessary.

## Figures and Tables

**Table 1:** Socio-demographic characteristic of the study group.

Variable		Total (*N* = 108)
Age (years)	Average ± SD	52.29 ± 3.85
	Me	52
	Quartiles	49–55
Place of residence	City	89 (82.41%)
	Village	19 (17.59%)
Workplace	Hospital	53 (49.07%)
	PHC	55 (50.93%)
Employment	Full-time job	107 (99.07%)
	Part-time job	1 (0.93%)
Work system	One shift work	52 (48.15%)
	Shift work and night duty	56 (51.85%)
Education	Basic nursing education	39 (36.11%)
	Bachelor of nursing	21 (19.44%)
	Nursing (master’s degree)	48 (44.44%)
Additional qualifications	No	20 (18.52%)
	Yes	88 (81.48%)
Specialization	No	62 (57.41%)
	Yes	46 (42.59%)
Qualification course	No	78 (72.22%)
	Yes	30 (27.78%)
A specialist course	No	71 (65.74%)
	Yes	37 (34.26%)
Training course	No	72 (66.67%)
	Yes	36 (33.33%)
Self-assessment of health condition	Very good	16 (14.81%)
	Good	68 (62.96%)
	I have no opinion	18 (16.67%)
	Bad	6 (5.56%)
	Very bad	0 (0.00%)
Participation in preventive examinations	No	65 (60.19%)
	Yes	43 (39.81%)
Chronic diseases	No	66 (61.11%)
	Yes	42 (38.89%)

Me—median; SD—standard deviation, PHC—Primary Health Care.

**Table 2:** Values of individual parameters in the study group.

Variable	Average ± SD	Me	Quartiles
FM (kg)	28.23 ± 10.07	27.5	20.21–34.07
FFM (kg)	41.98 ± 4.68	41.72	39.09–44.92
A/G	1 ± 0.21	1.02	0.87–1.15
BMI (kg/m^2^)	27.56 ± 5.87	27	23.05–30.33
TC (mg/dL)	220.34 ± 40.37	223	190.75–247
HDL (mg/dL)	57 ± 15.29	56	47–65
LDL (mg/dL)	135.35 ± 38.63	131.5	111–161.75
Triglycerides (mg/dL)	153.24 ± 77.57	137	98.5–203
Glucose (mg/dL)	95.08 ± 14.13	94	88–103
SBP (mmHg)	132 ± 19.49	128	119.75–147.25
DBP (mmHg)	79.54 ± 9.71	80	72.75–88

FM—fat mass, FFM—fat free mass, A/G—Android-to-gynoid ratio, BMI—Body mass index, TC—Total cholesterol, HDL—high-density lipoprotein, LDL—low-density lipoprotein, SBP—systolic blood pressure, DBP—diastolic blood pressure, Me—median, SD—standard deviation.

**Table 3:** Category of variables in the study group.

Variable		Total (*N* = 108)
Body mass category	Underweight	2 (1.85%)
	Normal body mass	35 (32.41%)
	Overweight	40 (37.04%)
	Class I obesity	21 (19.44%)
	Class II obesity	6 (5.56%)
	Class III obesity	4 (3.70%)
Blood pressure	Normal	57 (52.78%)
	Elevated	8 (7.41%)
	High blood pressure Stage 1	35 (32.41%)
	High blood pressure Stage 2	7 (6.48%)
	Hypertensive crisis	1 (0.93%)
Glucose	Hypoglycemia	1 (0.93%)
	Normal	71 (65.74%)
	Pre-diabetes	34 (31.48%)
	Diabetes	2 (1.85%)
Total cholesterol	Below standard	5 (4.63%)
	Normal	22 (20.37%)
	Elevated	81 (75.00%)
LDL	Normal	34 (31.48%)
	Elevated	74 (68.52%)
HDL	Below standard	11 (10.19%)
	Normal	97 (89.81%)
Triglycerides	Below standard	1 (0.93%)
	Normal	59 (54.63%)
	Elevated	48 (44.44%)

HDL—high-density lipoprotein, LDL—low-density lipoprotein.

**Table 4:** Health behavior of the respondents based on the Health Behavior Inventory (HBI) questionnaire.

HBI—Number of Points Nurses	Interpretation	*N*	%
24–77	Low	35	32.41%
78–91	Average	48	44.44%
92–120	High	25	23.15%

**Table 5:** Physical activity of the respondents based on the International Physical Activity Questionnaire.

MET	Interpretation	*N*	%
0–600	Insufficient activity	43	39.81%
600–1500	Activity sufficient	28	25.93%
>1500	High activity	35	32.41%
---	Questionnaire not completed	2	1.85%

MET—Metabolic Equivalent of Work2.2. The Findings.

**Table 6:** The occurrence of metabolic syndrome and its component.

Variable	Group	N	MS	MS Component				
				TG	HDL	Glucose	Blood Pressure	Abdominal Obesity
Total		108	38.9%	44.4%	30.6%	13.0%	47.2%	79.6%
Age	<50 years	39	20.5%	30.8%	23.1%	5.1%	35.9%	59.0%
	51–55 years	45	51.1%	48.9%	33.3%	17.8%	53.3%	91.1%
	>55 years	24	45.8%	58.3%	37.5%	16.7%	54.2%	91.7%
Place of residence	City	89	39.3%	43.8%	27.0%	13.5%	49.4%	78.7%
	Village	19	36.8%	47.4%	47.4%	10.5%	36.8%	84.2%
Workplace	Hospital	53	32.1%	41.5%	30.2%	9.4%	41.5%	77.4%
	PHC	55	45.5%	47.3%	30.9%	16.4%	52.7%	81.8%
Employment	Full-time job	52	38.5%	44.2%	32.7%	9.6%	50.0%	80.8%
	Part-time job	56	39.3%	44.6%	28.6%	16.1%	44.6%	78.6%
Education	One shift work	39	48.7%	48.7%	33.3%	17.9%	64.1%	84.6%
	Shift work and night duty	21	52.4%	61.9%	52.4%	9.5%	42.9%	90.5%
	Basic nursing education	48	25.0%	33.3%	18.8%	10.4%	35.4%	70.8%
Additional qualifications	No	20	45.0%	50.0%	25.0%	15.0%	60.0%	75.0%
	Yes	88	37.5%	43.2%	31.8%	12.5%	44.3%	80.7%
Specialization	No	62	43.5%	50.0%	33.9%	12.9%	56.5%	77.4%
	Yes	46	32.6%	37.0%	26.1%	13.0%	34.8%	82.6%
Qualification course	No	78	38.5%	44.9%	29.5%	14.1%	47.4%	80.8%
	Yes	30	40.0%	43.3%	33.3%	10.0%	46.7%	76.7%
A specialist course	No	71	39.4%	45.1%	32.4%	9.9%	52.1%	81.7%
	Yes	37	37.8%	43.2%	27.0%	18.9%	37.8%	75.7%
Training course	No	72	34.7%	41.7%	27.8%	13.9%	44.4%	80.6%
	Yes	36	47.2%	50.0%	36.1%	11.1%	52.8%	77.8%
Self-assessment of health condition	I have no opinion, bad	24	37.5%	45.8%	25.0%	4.2%	41.7%	91.7%
	Good	68	41.2%	44.1%	35.3%	19.1%	50.0%	80.9%
	Very good	16	31.2%	43.8%	18.8%	0.0%	43.8%	56.2%
Participation in preventive examinations	No	65	38.5%	46.2%	30.8%	12.3%	47.7%	72.3%
	Yes	43	39.5%	41.9%	30.2%	14.0%	46.5%	90.7%
Chronic diseases	No	66	36.4%	43.9%	30.3%	12.1%	45.5%	77.3%
	Yes	42	42.9%	45.2%	31.0%	14.3%	50.0%	83.3%
BMI	Underweight and normal body mass	37	13.5%	18.9%	18.9%	8.1%	40.5%	43.2%
	Overweight	40	45.0%	52.5%	32.5%	20.0%	40.0%	97.5%
	Obesity	21	57.1%	66.7%	42.9%	4.8%	61.9%	100.0%
	Class II and III obesity	10	70.0%	60.0%	40.0%	20.0%	70.0%	100.0%
HBI	Low	35	34.3%	42.9%	34.3%	17.1%	40.0%	77.1%
	Average	48	50.0%	47.9%	33.3%	10.4%	58.3%	83.3%
	High	25	24.0%	40.0%	20.0%	12.0%	36.0%	76.0%
IPAQ	Insufficient activity	43	46.5%	46.5%	41.9%	18.6%	58.1%	83.7%
	Activity sufficient	28	42.9%	53.6%	28.6%	7.1%	53.6%	85.7%
	High activity	35	28.6%	34.3%	20.0%	11.4%	31.4%	68.6%

MS—metabolic syndrome, BMI—body mass index, TG—triglycerides, HDL—high-density lipoprotein, PHC—Primary Health Care, HBI—the Health Behavior Inventory, IPAQ—International Physical Activity Questionnaire.

**Table 7:** The influence of individual variables on the occurrence of MS and MS components.

Variable	Group	OR for MS and Components					
		MS	TG	HDL	Glucose	Blood Pressure	Abdominal Obesity
Age	<50 years	1	1	1	1	1	1
	51–55 years	4.051 *	2.152	1.667	4	2.041	7.13 *
	>55 years	3.279 *	3.15 *	2	3.7	2.11	7.652 *
Place of residence	City	1	1	1	1	1	1
	Village	0.9	1.154	2.437	0.755	0.597	1.448
Workplace	Hospital	1	1	1	1	1	1
	PHC	1.765	1.263	1.035	1.878	1.572	1.317
Work system	One shift work	1	1	1	1	1	1
	Shift work and night duty	1.035	1.017	0.824	1.8	0.806	0.873
Education	Basic nursing education	1	1	1	1	1	1
	Bachelor of nursing	1.158	1.711	2.2	0.481	0.42	1.727
	Nursing (master’s degree)	0.351 *	0.526	0.462	0.532	0.307 *	0.442
Additional qualifications	No	1	1	1	1	1	1
	Yes	0.733	0.76	1.4	0.81	0.531	1.392
Specialization	No	1	1	1	1	1	1
	Yes	0.627	0.586	0.689	1.012	0.411 *	1.385
Qualification course	No	1	1	1	1	1	1
	Yes	1.067	0.939	1.196	0.677	0.97	0.782
A specialist course	No	1	1	1	1	1	1
	Yes	0.935	0.929	0.773	2.133	0.559	0.697
Training course	No	1	1	1	1	1	1
	Yes	1.682	1.4	1.47	0.775	1.397	0.845
Self-assessment of health condition	I have no opinion, bad	1	1	1	1	1	1
	Good	1.167	0.933	1.636	5.436	1.4	0.385
	Very good	0.758	0.919	0.692	<0.001	1.089	0.117 *
Participation in preventive examinations	No	1	1	1	1	1	1
	Yes	1.046	0.84	0.975	1.155	0.954	3.734 *
Chronic diseases	No	1	1	1	1	1	1
	Yes	1.312	1.054	1.031	1.208	1.2	1.471
BMI	Underweight and normal body mass	1	1	1	1	1	1
	Overweight	5.236 *	4.737 *	2.063	2.833	0.978	51.187 *
	Obesity	8.533 *	8.571 *	3.214	0.567	2.383	>1000
	Class II and III obesity	14.933 *	6.429 *	2.857	2.833	3.422	>1000
HBI	Low	1	1	1	1	1	1
	Average	1.917	1.227	0.958	0.562	2.1	1.481
	High	0.605	0.889	0.479	0.659	0.844	0.938
IPAQ	Insufficient activity	1	1	1	1	1	1
	Activity sufficient	0.863	1.327	0.556	0.337	0.831	1.167
	High activity	0.46	0.6	0.347 *	0.565	0.33 *	0.424

OR—odds ratio, MS—metabolic syndrome, BMI—body mass index, TG—triglycerides, HDL—high-density lipoprotein, PHC—Primary Health Care, HBI—the Health Behavior Inventory, IPAQ—International Physical Activity Questionnaire, *p*—*p*-value, indicate significant values (*p* < 0.05), *—indicate significant values (*p* < 0.05).

**Table 8:** Statistical significance of factors in the regression model for metabolic syndrome.

Variable	Group	OR for MS and Components					
		MS	TG	HDL	Glucose	Blood Pressure	Abdominal Obesity
Age	<50 years	1	1	1	1	1	1
	51–55 years	2.884	1.712	1.203	3.445	2.312	10.877
	>55 years	1.532	3.407	0.911	17.682	1.099	1.924
Place of residence	City	1	1	1	1	1	1
	Village	0.377	0.815	3.198	0.225	0.368	0.416
Workplace	Hospital	1	1	1	1	1	1
	PHC	2.733	1.327	0.494	15.493 *	1.337	1.529
Work system	One shift work	1	1	1	1	1	1
	Shift work and night duty	1.517	1.39	0.549	5.398	1.027	2.958
Education	Basic nursing education	1	1	1	1	1	1
	Bachelor of nursing	1.898	1.939	5.68 *	0.75	0.473	0.257
	Nursing (master’s degree)	0.415	1.121	0.639	0.872	0.312	0.062
Specialization	No	1	1	1	1	1	1
	Yes	0.574	0.33	0.662	1.286	0.468	4.617
Qualification course	No	1	1	1	1	1	1
	Yes	0.815	0.993	1.255	0.075	1.571	1.461
A specialist course	No	1	1	1	1	1	1
	Yes	1.051	1.371	0.49	24.926 *	0.419	1.949
Training course	No	1	1	1	1	1	1
	Yes	3.343 *	1.529	1.677	0.721	1.569	0.251
Self-assessment of health condition	I have no opinion, bad	1	1	1	1	1	1
	Good	2.324	1.631	2.782	18.261	2.151	0.294
	Very good	2.527	2.897	1.215	<0.001	4.641	0.004
Participation in preventive examinations	No	1	1	1	1	1	1
	Yes	0.567	0.472	1.246	0.739	0.725	2.595
Chronic diseases	No	1	1	1	1	1	1
	Yes	2.051	0.947	0.884	0.332	2.091	0.101
BMI	Underweight and normal body mass	1	1	1	1	1	1
	Overweight	8.58 *	7.441 *	2.158	9.211	1.073	296.821 *
	Obesity	8.085 *	7.194 *	3.562	0.436	1.489	>1000
	Class II and III obesity	16.505 *	5.132	2.455	1.69	1.96	>1000
HBI	Low	1	1	1	1	1	1
	Average	1.597	1.018	0.899	0.516	2.218	1.359
	High	0.246	0.704	0.293	0.513	0.647	2.403
IPAQ	Insufficient activity	1	1	1	1	1	1
	Activity sufficient	0.972	2.296	0.75	0.44	1.356	0.265
	High activity	0.436	0.75	0.292	0.657	0.245 *	0.284

OR—odds ratio, MS—metabolic syndrome, BMI—body mass index, TG—triglycerides, HDL—high-density lipoprotein, PHC—Primary Health Care, HBI—the Health Behavior Inventory, IPAQ—International Physical Activity Questionnaire, *p*—*p*-value, indicate significant values (*p* < 0.05), *—indicate significant values (*p* < 0.05).

## Data Availability

The data presented in this study are available on reasonable request from the corresponding author. The data are not publicly available due to restrictions e.g. their containing information that could compromise the privacy of research participants.
